# Interrelationships between social exclusion, mental health and wellbeing in adolescents: insights from a national *Youth Survey*

**DOI:** 10.1017/S2045796024000878

**Published:** 2025-01-13

**Authors:** K. Filia, S. M. Teo, N. Brennan, T. Freeburn, D. Baker, V. Browne, A. Watson, J. Menssink, A. Prasad, E. Killackey, P. D. McGorry, S. M. Cotton, C. X. Gao

**Affiliations:** 1 Centre for Youth Mental Health, The University of Melbourne, Parkville, VIC, Australia; 2 Orygen, Parkville, VIC, Australia; 3 Mission Australia, Sydney, NSW, Australia; 4 Turner Institute for Brain and Mental Health, School of Psychological Sciences, Monash University, Melbourne, VIC, Australia; 5 Department of Epidemiology and Preventative Medicine, School of Public Health and Preventive Medicine, Monash University, Melbourne, VIC, Australia

**Keywords:** adolescent mental health, prevention and early intervention, social determinants, social exclusion, youth survey, youth wellbeing

## Abstract

**Aims:**

Adolescence is a critical developmental phase during which young people are vulnerable to the experiences of mental ill-health and social exclusion (consisting of various domains including education and employment, housing, finances and social supports and relationships). The aims of this study were to (i) obtain an understanding of the relationships between social exclusion, mental health and wellbeing of young people; and (ii) identify potentially modifiable targets, or population groups that require greater or targeted supports.

**Methods:**

Data were obtained from the Mission Australia 2022 *Youth Survey*, Australia’s largest annual population-wide survey of young people aged 15–19 years (*n* = 18,800). Participants’ experiences of social exclusion in different domains were explored (e.g., prevalence, co-occurrence and controlling for differences in demographic characteristics). Multivariable linear regression models were used to map the relationships between social exclusion domains and mental health and wellbeing, controlling for confounding factors where necessary.

**Results:**

Sixty per cent of all young people experienced social exclusion in at least one domain, 25% in multiple. Young people who identified as gender diverse, Indigenous, living in a remote/rural or socio-economically disadvantaged area and with a culturally diverse background were more likely to report social exclusion. A strong association was seen between all domains of social exclusion and poor mental health (e.g., higher psychological distress and loneliness, reduced personal wellbeing, reduced sense of control over their life and a more negative outlook on the future). Notably, difficulties in socialising and obtaining social support were critical factors linked to increased psychological distress and reduced wellbeing.

**Conclusions:**

Findings underscore the need to address multiple domains of social exclusion concurrently, and in collaboration with youth mental healthcare. Prevention efforts aimed at early identification and intervention should be prioritised to support young people vulnerable to social exclusion. Screening approaches are needed to identify individuals and groups of young people in need of support, and to facilitate care coordination across multiple providers.

Social exclusion is an overarching social determinant of mental health (Braveman and Gottlieb, 2014), encompassing impairment in several functional domains including employment, education, finances, housing, neighbourhood and inaccessibility to services, which act independently and synergistically as social determinants of mental health (Filia *et al.*, 2018, 2019). Social *inclusion* refers to a sense of connectedness in interpersonal relations, as well as having the ability and opportunities for active participation in the aforementioned domains. These domains are essential to conceptualising social inclusion and exclusion, as identified in thematic analysis of literature by Filia *et al.* (2018) and supported by a consensus study of people with and without a lived experience of mental ill-health (Filia *et al.*, 2019).

In Australia, social exclusion affects approximately 25% of the population, with an estimated 1.2 million Australians (4.7%) experiencing profound, or deep, social exclusion that traverses multiple domains (Brotherhood of St Laurence and Melbourne Institute of Applied Economic and Social Research (MIAESR) 2020). It is disproportionately experienced by people with mental ill-health (Wang *et al.*, 2018), and is often exacerbated by clinical symptomatology, resulting in a complex and cyclical relationship that can be difficult to break (Filia *et al.*, 2018). This is further complicated by interrelationships between the different domains of social exclusion, with disadvantage in one domain contributing to run-on effects in others. Conversely, these complex interrelationships mean that improvements in one domain can create positive change in others.

Adolescence is an important life stage during which brain development and interactions with the social environment form the foundations for lifelong wellbeing and socio-economic productivity (Patton *et al.*, 2016). It is also a period during which young people are most vulnerable to experiences of mental ill-health and the onset of mental disorders (Caspi *et al.*, 2020; Solmi *et al.*, 2022). This timing can adversely impact social and occupational functioning (Iorfino *et al.*, 2022), derailing activities such as the formation of independent social and romantic relationships, completing formal education, entering the workforce and taking increasing autonomy over finances, housing and lifestyle choices. It can be difficult to recover from disruptions to these activities during adolescence (Patton *et al.*, 2016). Unsurprisingly, an adolescent’s vulnerability to mental health difficulties increases their vulnerability to social exclusion (Berry and Greenwood, 2018).

Social exclusion provides a useful framework to consider the dynamic interplay between contributors to mental ill-health, highlighting the impact of multiple, or intersecting forms of disadvantage such as low income, poor housing, unemployment or underemployment, and/or a lack of social participation (Brown *et al.*, 2016). As per the concept of intersectionality, the co-occurrence of these forms of disadvantage exacerbates negative impacts, creating more complex or challenging circumstances (Seng *et al.*, 2012). Recently, we reported that in a large sample of help-seeking young people in Australia, the compounding experience of multiple forms of disadvantage (e.g., unstable housing and disengagement from education/employment) was associated with significantly greater rates of distress, suicidal ideation and substance use in those who had experienced exclusion across multiple domains compared to those who had experienced unidimensional or no social exclusion (Filia, Menssink, *et al.* 2022).

Understanding social determinants of mental ill-health and, in particular social exclusion as a key social determinant, is important because these are modifiable factors; as circumstances change, so too does their impact on mental health. These factors are therefore potentially amenable to intervention (Braveman and Gottlieb, 2014). Adolescence, as a critical stage during which vulnerability to mental ill-health is high, and foundational activities for life-long social inclusion occur (Berry and Greenwood, 2018), presents an opportune period for targeted interventions. Following the onset of mental ill-health, early intervention approaches promoting functional recovery and preventing further decline are crucial to reducing the impact of mental ill-health on functioning and quality of life (McGorry *et al.*, 2022). A better understanding of how social determinants impact mental health during adolescence, and the interplay between specific determinants and early-life social inclusion is imperative for minimising the adverse long-term effects on mental ill-health (Braveman and Gottlieb, 2014).

## Current study

Utilising the most recent data from the largest annual survey of young people in Australia, the 2022 Mission Australia *Youth Survey* (*n* = 18,800), the aims of the current study were twofold: (i) to obtain a better understanding of the relationships between social exclusion domains, mental health and wellbeing of young people aged 15–19 years; and (ii) identify potentially modifiable targets, or population groups requiring greater or targeted supports.

Given the complex effects of social exclusion, we examined a comprehensive set of mental health and wellbeing variables – psychological distress, subjective wellbeing, perceived mental health, control over one’s life, loneliness, feelings about the future and presence of a mental health condition. These are all particularly relevant during adolescence, during which relationships between these aspects of mental health and wellbeing and social exclusion can impact lifelong trajectories. For instance, hope and a positive outlook toward the future can help buffer against the adverse effects of social exclusion, such as unstable living conditions and family conflict. Conversely, loneliness, psychological distress and lack of control have well-documented associations with negative outcomes, including anxiety, depression and impaired cognitive and physical health (Almeida *et al.*, 2021; Goosby *et al.*, 2013; Mushtaq *et al.*, 2014). By examining this specific set of variables, our study aims to provide a nuanced understanding of the relationships between social exclusion and youth mental health, and to highlight potential intervention points to support young people facing multiple forms of disadvantage.

## Methods

### Study design and participants

Young people aged 15–19 years were recruited from each Australian state and territory between 6 April and 31 August 2022. The Mission Australia *Youth Survey* team engaged with schools, collaborating bodies, community organisations and local government services to promote the survey, as well as via social media, Mission Australia services and the Mission Australia website.

Participants completed the self-report measure online or by paper, following an informed consent script, without reimbursement. All procedures were approved by the University of Melbourne Human Research and Ethics Committee (#2022-22721-32663), State and Territory Education Departments and Catholic Education offices.

### Measures

#### The Mission Australia Youth Survey

Mission Australia, a large national charity, has conducted annual surveys of young people in Australia since 2002. The 2022 *Youth Survey* consisted of 45 question sets, including embedded validated measures covering a range of topics including education and employment, social and family support, community engagement, mental health, general wellbeing and the values and concerns of young people. A description of items and an overview of responses can be found elsewhere (Leung *et al.*, 2022). To address our aims, we utilised a subset of variables, with details below and in Table 1.

**Table 1.** Variables, classifications and items from the 2022 *Youth Survey*

Variable Social exclusion	Classifications
Relational difficulties	Participants were deemed as experiencing relational difficulties if they had answered *Yes* to both of the following two questions: *Do you find it hard to fit in and socialise with everyone else (at school, work or socially)* and *Do you find it hard to turn to friends and family if you need help?*
Housing challenges	Participants were determined as experiencing housing challenges if they had answered *Yes* to any of the following: *Have you experienced a time when you had no fixed address or lived in a refuge or transitional accommodation within the last year? Within the last year, have you spent time away from home because you felt you couldn’t go back?* or *In the past year, have you ever worried about having a safe place to stay?*
Financial hardships	Participants were determined as experiencing financial hardships if they had answered *Yes, Mission Australia, Yes, a different charity or foundation*, or *No, but I needed support or assistance* to the question *In the past year, have you and/or your family received support or assistance from a charity or foundation?* OR answered *Could not pay bills or car expenses, Could not pay rent/mortgage, Gone without a meal, Could not afford school supplies or go on school excursions*, or *Sought financial help from family, friends or a charity* to the question *In the past year, have you and/or your family experienced any of the following because of money concerns?*
Edu-employment issues	Edu-employment issues were determined by participants current vocational activity or lack thereof and categorised as *not engaged in employment, education or training (NEET), or not satisfied with studies*. The latter category was obtained by responses of *Very dissatisfied* or *Dissatisfied* to the question *How satisfied are you with your studies?*
**Mental health and wellbeing**	
Psychological distress	Psychological distress was measured using the Kessler Psychological Distress Scale – 6 item version (K6) (Kessler *et al.* 2003). K6 consists of six questions used to measure non-specific psychological distress including items related to nervousness, hopelessness, restlessness, sadness, feeling that everything was an effort and worthlessness. Respondents were classified into two groups: *No probable serious mental illness* (<18), and *probable serious mental illness* (>18) (Furukawa et al. 2003; Kessler et al. 2010).
Wellbeing	Wellbeing was assessed using the School Children version of the Personal Wellbeing Index (PWI-SC; Cummins and Lau, 2005; Tomyn and Cummins, 2011). The PWI-SC consists of seven items related to how happy the young person feels with respect to various aspects of their life including their health, the things they have, how they get along with others, and how safe they feel. Scores were converted to a 100-point scale and rounded to the nearest integer. Respondents were classified into three groups according to the PWI-SC manual (Cummins and Lau 2005): *Personal wellbeing likely to be challenged* (<50), *personal wellbeing likely to be compromised* (51 − 69), and *likely experiencing a normal level of wellbeing* (>70).
Perceived mental health & wellbeing	Participants were asked to rate their mental health and wellbeing, with responses on a scale of 1−5 (poor, fair, good, very good, excellent).
Control over life	Participants were asked to indicate how much control they felt they had over their lives, with responses: *no, almost no, some, mostly in* and *complete control.* We combined *no control* and *almost no control* into a single category.
Loneliness	Participants were asked to indicate how much of the time they felt lonely, with responses: *all of the time, most of the time, some of the time, a little of the time, none of the time*. We combined *all of the time* and *most of the time* into a single category.
Feelings about future	Participants were asked to rate their outlook for the future: *How would you describe your feelings when you think about the future?* with a five-point scale from *very negative* to *very positive*. Responses were categorised into ‘very negative/negative’, “neither negative nor positive” and ‘very positive/positive’.
Mental health condition	Do you identify as a person with a mental health condition?
**Demographics and correlates of social exclusion and mental health**	
Gender	Self-identified gender options included *female, male, non-gendered, non-binary gender, transgender, not listed* and *prefer not to say.* Non-gendered, non-binary gender, transgender, and not listed were grouped as ‘gender diverse’.
Age group	15−17 or 18−19 years
Indigenous status	Do you identify as Aboriginal and/or Torres Strait Islander?
Geographic locality	Locality information was obtained from the Australian Bureau of Statistics (ABS) by matching participants’ residential postcodes to one of five categories: *Major Cities of Australia, Inner Regional Australia, Outer Regional Australia, Remote Australia, Very Remote Australia*. The latter three are combined in our analyses as ‘Outer regional, remote or very remote’.
Index of Relative Socio-economic Advantage and Disadvantage	Index of Relative Socio-economic Advantage and Disadvantage (IRSAD) information were obtained from the Australian Bureau of Statistics (ABS) by matching participants’ residential postcodes. IRSAD is an area-based socio-economic status measure that summarises census information about the economic and social conditions of residents, including measures of both relative advantage and disadvantage (higher deciles indicate relative lack of disadvantage and greater advantage in general) (ABS 2023).
Language other than English	Do you speak a language other than English at home?

##### Social exclusion

To operationalise social exclusion using variables from the survey, we created indices of four domains of social exclusion previously identified as key (Filia, Gao, *et al.* 2022): relational difficulties, financial hardship, housing challenges and edu-employment issues. Items were selected based on their suitability for capturing each domain’s essential aspects, and alignment with the *Youth Survey*’s structure and goals rather than adhering to a predefined set of items.

##### Mental health and wellbeing

Measures included the six-item Kessler Psychological Distress Scale (K6) (Kessler et al., 2002), and the Personal Wellbeing Index – School Children version (PWI-SC) (Cummins and Lau, 2005; Tomyn and Cummins, 2011). Participants were also asked to rate their mental health and wellbeing on a single item, report if they had a mental health condition, how they felt about the future, how often they felt lonely and the degree of control they felt over their lives.

##### Demographics and correlates of social exclusion and mental health

Demographic variables were used to aid in identifying population groups most vulnerable to social exclusion and/or mental ill-health, and acted as confounders in the analyses. They included age, gender, Indigenous status, speaking a language other than English at home and residential postcode. Postcodes were used to identify whether participants were living in metropolitan areas – rurally, regionally or in remote locations – and determine area-based socio-economic conditions using the Index of Relative Socio-economic Advantage and Disadvantage (IRSAD) obtained from the 2021 census (Australian Bureau of Statistics, 2023). These correlates were all selected as established risk factors associated with both mental health outcomes and social exclusion.

## Statistical methods

### Data cleaning and missing data

The data comprised 18,800 survey responders. Details of missing data are included in Table 2. The ‘mice’ R package was used to perform multiple imputation on missing data with 20 imputed datasets (Buuren and Groothuis-Oudshoorn, 2011) using the predictive mean matching method. Variables used for imputation included all variables in Table 1, as well as additional variables relating to mental health support, community support and connections, cultural and spiritual beliefs, activity groups and family relationships (see Table S1 in Supplementary Materials). Imputed data were used in regression modelling and results pooled using Rubin’s rule (Rubin, 1996). Correlations were assessed between potentially related measures, looking at relationships between the individual domains of the PWI-SC and social exclusion. Observed correlations were positive but weak, ranging from 0.10 to 0.35 (highest between the personal relationship domain of the PWI-SC and ‘relational difficulties’, as well as the personal safety domain and the PWI-SC and ‘housing challenges’). No further action was taken.

**Table 2.** Participant demographics for the total cohort (*N* = 18,800) as well as separately for each of the four social exclusion domains

		Housing challenges			Financial hardships			Relational difficulties			Edu-employment issues		
	Overall *N* = 18,800	No *n* = 15241	Yes *n* = 2801	*p*-value	No *n* = 14547	Yes *n* = 4253	*p*-value	No *n* = 15472	Yes *n* = 3328	*p*-value	No *n* = 11365	Yes *n* = 7148	*p*-value
**Gender**				<0.001			<0.001			<0.001			<0.001
Male	6037 (33.8%)	5013 (34.5%)	736 (27.7%)		4851 (35.1%)	1186 (29.5%)		5360 (36.4%)	677 (21.5%)		3523 (32.4%)	2415 (36.0%)	
Female	11042 (61.8%)	9021 (62.2%)	1641 (61.9%)		8482 (61.3%)	2560 (63.7%)		8886 (60.4%)	2156 (68.6%)		6987 (64.2%)	3907 (58.2%)	
Gender diverse	777 (4.4%)	476 (3.3%)	276 (10.4%)		504 (3.6%)	273 (6.8%)		465 (3.2%)	312 (9.9%)		374 (3.4%)	393 (5.9%)	
**Age group**				0.44			<0.001			0.777			0.66
15−17	17241 (91.7%)	14023 (92.0%)	2565 (91.6%)		13525 (93.0%)	3716 (87.5%)		14193 (91.8%)	3048 (91.6%)		10435 (91.8%)	6549 (91.7%)	
18−19	1553 (8.3%)	1213 (8.0%)	235 (8.4%)		1021 (7.0%)	532 (12.5%)		1274 (8.2%)	279 (8.4%)		927 (8.2%)	596 (8.3%)	
**ATSI**				<0.001			<0.001			<0.001			<0.001
Non-Indigenous	17431 (95.3%)	14628 (96.5%)	2502 (89.8%)		13605 (96.8%)	3826 (90.6%)		14315 (95.6%)	3116 (94.1%)		10435 (91.8%)	6549 (91.7%)	
Indigenous	851 (4.7%)	530 (3.5%)	285 (10.2%)		454 (3.2%)	397 (9.4%)		656 (4.4%)	195 (5.9%)		430 (3.9%)	404 (5.8%)	
**IRSAD Decile**, mean (SD)	7.1 (2.8)	7.3 (2.7)	6.4 (2.9)	<0.001	7.4 (2.7)	6.2 (2.9)	<0.001	7.2 (2.7)	6.7 (2.9)	<0.001	7.3 (2.7)	6.9 (2.8)	<0.001
**Remoteness**				<0.001			<0.001			<0.001			0.003
Major cities	12934 (71.9%)	10682 (72.8%)	1769 (66.2%)		10144 (73.0%)	2790 (68.2%)		10756 (72.7%)	2178 (68.2%)		8011 (73.0%)	4799 (70.9%)	
Inner regional	2977 (16.5%)	2385 (16.3%)	506 (18.9%)		2213 (15.9%)	764 (18.7%)		2373 (16.0%)	604 (18.9%)		1771 (16.1%)	1144 (16.9%)	
Outer regional, remote or very remote	2081 (11.6%)	1597 (10.9%)	399 (14.9%)		1545 (11.1%)	536 (13.1%)		1671 (11.3%)	410 (12.8%)		1185 (10.8%)	827 (12.2%)	
**Language spoken at home**				0.168			<0.001			0.695			<0.001
Only English	14601 (80.8%)	12183 (81.0%)	2190 (79.9%)		11351 (81.6%)	3250 (78.0%)		11944 (80.7%)	2657 (81.0%)		8994 (81.7%)	5411 (79.3%)	
Other(s)	3478 (19.2%)	2853 (19.0%)	551 (20.1%)		2563 (18.4%)	915 (22.0%)		2855 (19.3%)	623 (19.0%)		2017 (18.3%)	1414 (20.7%)	

*Note.* Missing data: 944 for gender, 808 IRSAD and remoteness, 758 housing challenges, 758 for K6, 721 for speaking a language other than English at home, 518 for Indigenous status, 287 for the edu-employment domain and 6 for age.

### Data analysis

All analyses were performed in R version 4.2.1 (2022-06-23). Simple descriptive analyses and graphical visualisations demonstrated the demographic characteristics of participants, and their health and wellbeing.

Characteristic differences were also compared between those who experienced social exclusion in individual domains versus those who did not using Pearson’s Chi-squared test for categorical variables and *t*-tests for continuous variables.

To explore the role and interplay of the different social exclusion domains on psychological distress (K6) and subjective wellbeing (PWI-SC), four sets of separate analyses were run for each outcome (K6 and PWI-SC as continuous variables), namely (1) multivariable linear regression models including each social exclusion domain separately as a risk factor, controlling for common confounders (partially adjusted model); (2) a multivariable linear regression model including all social exclusion domains as risk factors controlling for common confounders (fully adjusted model); (3) multivariable linear regression models testing whether any interactions existed between social exclusion domains (interaction model); and (4) multivariable linear regression models with the number of social exclusion domains as the risk factor.

## Results

### Demographics and social exclusion

Demographic characteristics are included in Table 2. Participants who identified as gender diverse, were older (aged 18–19 vs. 15–17 years), were Indigenous, were living in areas of lower IRSAD – regionally or remotely and spoke a language other than English at home were more likely to report social exclusion.

**Figure 1.**
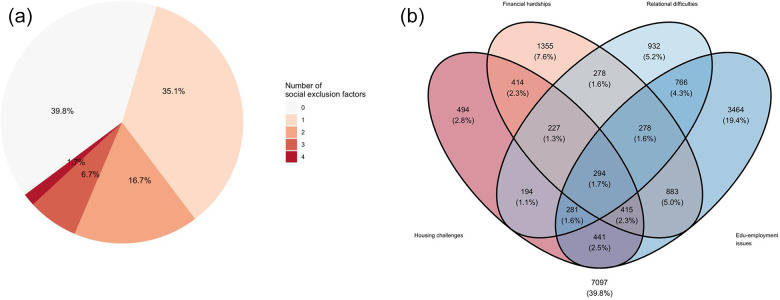
The proportion of social exclusion reported by sample by each domain, and proportions in multiple domains. (a) Pie chart of the number of social exclusion domains participants reported, (b) Venn diagram portraying experiences of social exclusion in multiple and overlapping domains

Looking at single domains, 39% reported edu-employment issues, 23% financial hardship, 18% experienced relational difficulties and 16% experienced housing challenges. Sixty per cent of the sample experienced exclusion in at least one domain, and 25% in multiple (Fig. 1a). There was a strong intersectional component (Fig. 1b); the four domains had similar overlaps with one other, except for the edu-employment domain with slightly higher exclusive overlaps with relational (4.3%) and financial hardship (5%) domains.

### Mental health and wellbeing

**Figure 2.**
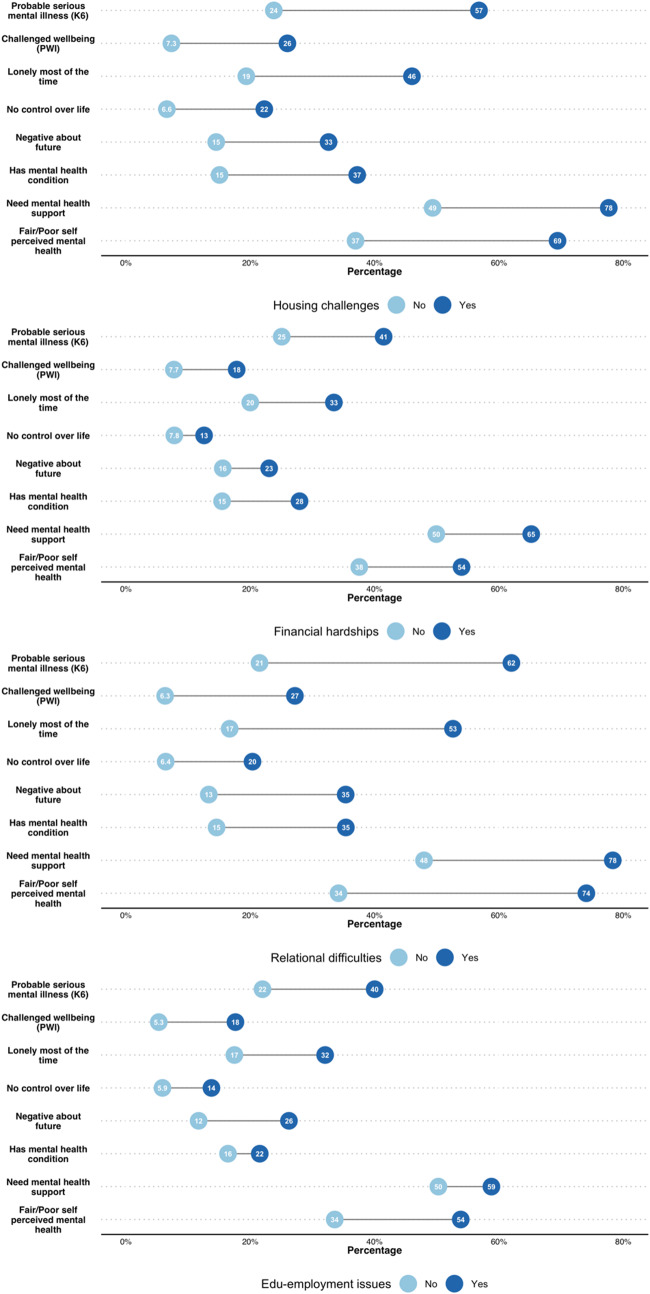
Proportion of participants who experienced mental health and wellbeing issues by social exclusion domain.

Participants who reported social exclusion in any domain were more likely to experience poorer mental health and lower wellbeing, more frequent feelings of loneliness, having no control over their lives and a negative outlook on the future (Fig. 2).

**Figure 3.**
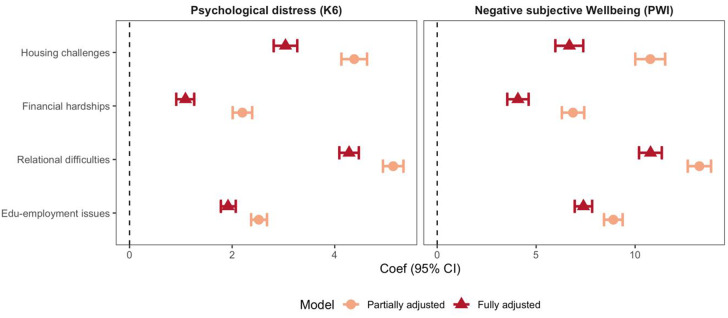
Multivariable linear regression for K6 and PWI-SC.

*Note.* All coefficients were estimated from multiple imputed multivariable linear regression models controlling for confounding factors including gender identity, age groups, Indigenous status, IRSAD decile, remoteness and whether the young person reported speaking a language other than English at home. In partially adjusted models, only one social exclusion domain was included, whereas in fully adjusted models, all social exclusion domains were included.

Univariate associations between social exclusion domains and psychological distress (K6) and wellbeing (PWI-SC) were retained in multivariable models controlling for demographic confounders. In partially adjusted models (one social exclusion domain included in each model; Fig. 3 and Table S2 in Supplementary Material), experiencing social exclusion in any domain was associated with a 2–5 score increase in K6. Relational difficulties were found to have the largest effect (5.14; 95%CI: 4.94, 5.34). When all social exclusion domains were jointly included in the fully adjusted model, point estimates were slightly smaller (Fig. 3). However, all social exclusion domains independently contributed to the increase in K6 scores, and the relational domain remained the critical factor differentiating psychological distress levels (4.28; 95% CI: 4.09, 4.47).

Similar results were identified for subjective wellbeing (PWI-SC; Fig. 3 and Table S2). Individual social exclusion domains were associated with a 6–14 score reduction in PWI-SC (partially adjusted model), reducing slightly in the fully adjusted model. When the two outcome total scores were standardised (Table S3), most social exclusion domains had a comparable or lower effect on PWI-SC except for the edu-employment domain, which was associated with further reduction in PWI-SC compared with K6 (0.33 SD increase in K6; 95% CI: 0.31, 0.36 vs. 0.44 SD decrease in PWI-SC; 95% CI: 0.47, 0.42).

Potential interactions between individual social exclusion domains on the two outcomes were tested. Slightly lower than additive incremental effects of social exclusion across multiple domains were observed (negative interaction term for K6 models and positive interaction term for PWI-SC models; Tables S4 and S5). However, there remained a strong incremental effect of experiencing social exclusion across multiple domains on poor psychological distress and wellbeing (Table 3).

**Table 3.** Multivariable linear regression for K6 and PWI-SC using the number of social exclusion factors as the risk factor

	Psychological distress (K6)		Subjective wellbeing (PWI-SC)	
Risk factor Number of social exclusion factors	Coef (95% CI)	*p*-value	Coef (95% CI)	*p*-value
No	Ref		Ref	No
One	2.39 (2.22, 2.56)	<0.001	−7.45 (−7.94, −6.95)	<0.001
Two	5.10 (4.88, 5.32)	<0.001	−15.09 (−15.76, −14.43)	<0.001
Three	7.57 (7.26, 7.88)	<0.001	−21.43 (−22.36, −20.51)	<0.001
Four	9.05 (8.47, 9.63)	<0.001	−25.78 (−27.53, −24.03)	<0.001

*Note.* All coefficients were estimated from multiple imputed multivariable linear regression models controlling for confounding factors including gender identity, age groups, Indigenous status, IRSAD decile, remoteness and whether the young person reported speaking a language other than English at home.

## Discussion

This study was the first comprehensive analysis of the multiple domains of social exclusion and their relationships with mental health and wellbeing in young people, from the most recent dataset of the largest survey of young people aged 15–19 years nationwide. It provides, for the first time, the rates of social exclusion among young people in Australia, the significant overlap between domains of social exclusion and it highlights a strong association between social exclusion and poorer mental health. Our findings demonstrate the importance of considering multiple domains of social exclusion simultaneously; these domains often intersect and interact, leading to increased negative effects on mental health. These findings are particularly relevant in the context of the current youth mental health crisis, with an urgent need to understand drivers, and identify modifiable, malleable risk factors to inform effective interventions and reform (McGorry *et al.*, 2023).

By examining how various social determinants interact and relate to social exclusion, we identified specific population groups more vulnerable to social exclusion. Young people who identified as gender diverse, Indigenous, living in economically disadvantaged areas and spoke a language other than English at home were more likely to report social exclusion. This intersectional lens emphasises the need for targeted interventions that consider the unique, additional challenges faced by these vulnerable groups.

The relationship between social exclusion and youth mental health was evident, encompassing more than heightened psychological distress and lower personal wellbeing. When looking at individual social exclusion domains, young people who experienced exclusion in each reported increased loneliness, negative feelings about the future, lack of control over their lives, fair or poor overall perceived mental health and having a mental health condition. Associations between social exclusion, psychological distress and personal wellbeing were exacerbated when social exclusion was reported across multiple domains. These relationships continued to exist when analyses controlled for confounding factors, reinforcing the contribution of social exclusion on mental health and wellbeing, regardless of social circumstances, location and/or available resources. Considering the confounding factors (such as age, gender and cultural diversity) in interventions that directly target social exclusion remains crucial however – these young people represent priority groups with a high prevalence of social exclusion.

Our findings align with existing research that emphasises the impact of social exclusion on mental health and wellbeing, particularly during the critical developmental period of adolescence (Rickwood *et al.*, 2023). Relationships between social exclusion and correlates of mental health such as hope, control, loneliness and perceived wellbeing are important, particularly during the crucial juncture of adolescence. During adolescence, the presence of hope is instrumental in planning and working towards a positive future (Stoddard and Pierce, 2015). It has been recognised as a protective factor against aspects of social exclusion (e.g., unstable living conditions, poverty, family conflict) and mental ill-health (Bowers and Bowers, 2023). Similarly, loneliness is increasingly understood as an important component of mental health, displaying positive associations with anxiety, depression, self-harm, and suicidal ideation and behaviours (Mushtaq *et al.*, 2014), as well as negative impacts on academic achievement, planning and integrating into social circumstances (Goosby *et al.*, 2013). There are sustained effects of loneliness, extending beyond the time it is felt, such as delayed cognitive development, impaired cognitive functioning, greater psychosomatic complaints (e.g., headaches, nausea) and poorer overall health (e.g., high cortisol levels) (Almeida *et al.*, 2021; Goosby *et al.*, 2013).

Specific domains of social exclusion were observed as having greater impacts on mental health and wellbeing. This is an important finding, particularly with regards to informing resource allocation and development of novel interventions. While services to address any or all aspects of social exclusion are beneficial, it is helpful to know which aspects of social exclusion are most influential on mental health. The identification of relational difficulties as a particularly critical factor in differentiating psychological distress levels highlights the significance of peer relationships and social support networks in adolescents’ lives. These findings echo previous research that emphasises the importance of positive social interactions in mitigating the risk of poor mental health (Filia *et al.*, 2021; Peñate *et al.*, 2020; Scardera *et al.*, 2020).

### Implications and future directions

Several implications can be drawn from these findings, with the potential to improve immediate and long-term mental health outcomes for this population. With respect to mental health services, while social exclusion is recognised as a social determinant of mental health, it is not addressed as part of routine mental healthcare. Much of mental healthcare focuses on reducing mental health symptoms, a largely ‘medical model’ of care (Braveman and Gottlieb, 2014; Killaspy *et al.*, 2022), failing to address critical contributors to a person’s mental health (van Os *et al.*, 2019). However, employing a preventative framework and integrating strategies to address social exclusion as a core component of mental healthcare can improve health and wellbeing (Braveman and Gottlieb, 2014; Killaspy *et al.*, 2022), social isolation and loneliness, treatment engagement, illness management, long-term functional recovery (Dixon *et al.*, 2016; Goosby *et al.*, 2013) and reduce suicide risk (Motillon-Toudic *et al.*, 2022). This is particularly important for young people, as early intervention approaches may minimise potential impairment to existing structures of social inclusion (McGorry *et al.*, 2022; Shepherd and Parsonage, 2011).

The historical siloing of support services, such as housing, vocational, social and community groups is an ongoing challenge to delivering support to young people across multiple, intersecting domains. Indeed, targeted intervention in one domain independent of intersecting domains potentially limits the degree and sustainability of any immediate improvements for a young person. The key to developing integrated policies to create better support services is understanding the impact and power of the relationships between domains of social exclusion and developing collaborative approaches to addressing the needs of people affected by it. Along a similar vein, research thus far has often focused on a single determinant (e.g., social isolation) as outcomes. Our findings indicate that looking at data in such a way may limit the effectiveness and generalisability of interventions (e.g., should vocational interventions control for other factors such as housing or financial circumstances?). It is necessary to consider the global architecture that supports meaningful and active engagements in society.

Finally, identification of young people who require support – for either their mental health or social exclusion, considering each as determinants of one another (Rickwood *et al.*, 2023) – necessitates screening with multidimensional outcome measures, and referral pathways to integrated services. The first is possible, through population screening approaches, and school-based tools (acknowledging the limitation of young people who disengage from school early). We now need to advocate for the measurement of social exclusion and its multiple domains as part of routine outcome assessment in mental healthcare (Rickwood *et al.*, 2023), with referral pathways to appropriate community services. Similarly, we must encourage community support services to include mental health assessment as part of the service they provide to young people in need, with referral pathways in return.

### Strengths and limitations

The use of a large and diverse sample from the Mission Australia *Youth Survey* is a significant strength of this study. The comprehensive survey design allowed for the exploration of the four social exclusion domains, and their interrelationships with mental health. This attention to intersectionality recognises the compounding effects of multiple forms of disadvantage on mental health. Additionally, the study employed rigorous statistical methods, including multiple imputation to handle missing data, enhancing the validity of the findings.

Some limitations must be acknowledged. The cross-sectional data limited causal inferences, preventing us from establishing the direction of causality between social exclusion and mental health outcomes. Longitudinal studies with measures from young people and support workers could better provide valuable insights into the temporal and cyclical relationships between these variables and the full intersectionality of these constructs. Moreover, this would allow for examination of potential bidirectional nature of these constructs, illuminating whether wellbeing and distress may both be a determinant and consequence of social exclusion.

Additionally, the self-report nature of the survey may introduce response biases, and reliance on self-reported measures of mental health and wellbeing might not capture the full complexity of these constructs. It would have been advantageous to include a more comprehensive measure of social inclusion such as the F-SIM16 (Filia *et al.*, 2022a) to the *Youth Survey*; however, we acknowledge the limitation of including lengthy measures that increase respondent burden and reduce response rates in large surveys such as this. Finally, it is important to remain aware of potential item or content overlap in measures of constructs such as wellbeing and social exclusion. In this study, we only saw very small correlations between associated measures; however, it is important to consider this particularly confounding issue in future research.

## Conclusion

Our findings contribute to the growing body of literature recognising relationships between social exclusion and the mental health and wellbeing of young people. With the impact and relative contributions of domains differing with respect to mental health and wellbeing, this research highlights the need to consider the complexity of the lives of young people, particularly in the context of risk of mental ill-health and the role of social determinants such as social exclusion. Findings suggest that young people who experience social exclusion would be well-placed to receive or be monitored for mental health support, and that those who experience mental ill-health would be well-placed to receive wrap-around support to improve their social inclusion. Interventions should be multifaceted, encompassing various aspects of adolescents’ social, financial, housing and vocational circumstances as well as mental health to promote positive outcomes, both immediate and long-term.

## Supporting information

Filia et al. supplementary material Filia et al. supplementary material

## Data Availability

All R codes used for this analysis are shared in https://osf.io/h5fdg/. The data utilised here are owned by Mission Australia. Any data requests should be made directly to them at youthsurvey@missionaustralia.com.au.

## Supplementary material

The supplementary material for this article can be found at https://doi.org/10.1017/S2045796024000878.
