# Supplementary material for: Interrelationships between social exclusion, mental health and wellbeing in adolescents: insights from a national Youth Survey
Source: Epidemiol Psychiatr Sci. 2025 Jan 13;34:e5. doi: 10.1017/S2045796024000878 (PMC11735115; doi:10.1017/S2045796024000878)
Supplement: Filia et al. supplementary material [file S2045796024000878sup001.docx]

**Supplementary Material**

**Table S1.** Variables used in the imputation model, in addition to all variables listed in Table 1 of the manuscript.

| Variable | Definition |
| --- | --- |
| Mental health support | Participants were asked if they had ever felt they needed mental health support. |
| Things participants found hard to do | Participants were asked if they find it hard to:   1. Do everyday activities as young people their age usually do 2. Do things in public places with friends 3. Fit in and socialise with everyone else 4. Turn to friends and family if you need help 5. Turn to services/organisations if you need help 6. Make choices and feel independent 7. Travel around the community   They were able to select all that apply. |
| Participation in groups/activities | Participants were asked if they have been involved in the following groups/activities:   1. Arts/cultural/music groups/activities 2. Environmental groups/activities 3. Political groups/activities 4. Religious groups/activities 5. Sports (as a participant) 6. Sports (as a spectator) 7. Student leadership groups/activities 8. Volunteer work 9. Youth groups/activities   They were able to select all that apply. |
| Beliefs | Participants were asked if they have spiritual or cultural beliefs. Choices include: Both spiritual and cultural beliefs, spiritual beliefs only, cultural beliefs only, or no beliefs. |
| Family relationships | Participants were asked to rate their family’s ability to get along with one another, with responses on a scale of 1-5 (poor, fair, good, very good, excellent). |
| Community connections | Participants were asked to rate the extent to which they agree or disagree with the following statements:   1. I am proud to be part of my community 2. Young people in my community have a say on issues that matter to them 3. My community has the things that I need to have a positive and thriving future   Responses were on a scale of 1-5 (Strongly disagree, disagree, mixed feelings, agree, strongly agree). |

**Table S2.** Standardised Coefficients (β) and 95% CI of β for partially and fully-adjusted multivariable linear regression models with the four social exclusion domains predicting K6 and PWI-SC

|  | Partially adjusted | | Fully adjusted | |
| --- | --- | --- | --- | --- |
|  | **Coef (95% CI)** | **p-value** | **Coef (95% CI)** | **p-value** |
| Psychological distress (K6) | | | | |
| Housing challenges | 4.38 (4.13, 4.63) | <0.001 | 3.04 (2.81, 3.27) | <0.001 |
| Financial hardships | 2.20 (2.01, 2.39) | <0.001 | 1.09 (0.91, 1.26) | <0.001 |
| Relational difficulties | 5.14 (4.94, 5.34) | <0.001 | 4.28 (4.09, 4.47) | <0.001 |
| Edu-employment issues | 2.52 (2.37, 2.68) | <0.001 | 1.92 (1.78, 2.07) | <0.001 |
| Subjective wellbeing (PWI-SC) | | | | |
| Housing challenges | -10.77 (-11.52, -10.01) | <0.001 | -6.68 (-7.38, -5.97) | <0.001 |
| Financial hardships | -6.86 (-7.43, -6.30) | <0.001 | -4.08 (-4.62, -3.54) | <0.001 |
| Relational difficulties | -13.25 (-13.84, -12.66) | <0.001 | -10.78 (-11.35, -10.20) | <0.001 |
| Edu-employment issues | -8.90 (-9.37, -8.43) | <0.001 | -7.39 (-7.83, -6.95) | <0.001 |

**NB**. All coefficients were estimated from multiple imputed multivariable linear regression models controlling for confounding factors including gender identity, age groups, Indigenous status, IRSAD decile, remoteness, and whether the young person reported speaking a language other than English at home. In partially adjusted models only one social exclusion domain was included, whereas, in fully adjusted models, all social exclusion domains were included.

**Table S3.** Standardised Coefficients (β) and 95% CI of β for partially and fully-adjusted multivariable linear regression models with the four social exclusion domains predicting the K6 and PWI-SC

|  | Partially adjusted | | Fully adjusted | |
| --- | --- | --- | --- | --- |
|  | **Coef (95% CI)** | **p-value** | **Coef (95% CI)** | **p-value** |
| Psychological distress (K6) | | | | |
| Housing challenges | 0.76 (0.72, 0.80) | <0.001 | 0.53 (0.49, 0.57) | <0.001 |
| Financial hardships | 0.38 (0.35, 0.42) | <0.001 | 0.19 (0.16, 0.22) | <0.001 |
| Relational difficulties | 0.89 (0.86, 0.93) | <0.001 | 0.74 (0.71, 0.78) | <0.001 |
| Edu-employment issues | 0.44 (0.41, 0.47) | <0.001 | 0.33 (0.31, 0.36) | <0.001 |
| Subjective wellbeing (PWI-SC) | | | | |
| Housing challenges | -0.64 (-0.69, -0.60) | <0.001 | -0.40 (-0.44, -0.36) | <0.001 |
| Financial hardships | -0.41 (-0.44, -0.38) | <0.001 | -0.24 (-0.28, -0.21) | <0.001 |
| Relational difficulties | -0.79 (-0.83, -0.76) | <0.001 | -0.64 (-0.68, -0.61) | <0.001 |
| Edu-employment issues | -0.53 (-0.56, -0.50) | <0.001 | -0.44 (-0.47, -0.42) | <0.001 |

**NB.** All coefficients were estimated from multiple imputed multivariable linear regression models controlling for confounding factors including gender identity, age groups, Indigenous status, IRSAD decile, remoteness, and whether the young person reported speaking a language other than English at home. K6 and PWI-SC total scores were standardised to provide comparable interpretation. In partially adjusted models only one social exclusion domain was included, whereas, in fully adjusted models, all social exclusion domains were included.

**Table S4.** Multivariable linear regression for K6 (fully adjusted) with interaction terms.

|  | Coef (95% CI) | p-value |
| --- | --- | --- |
| Model with the interaction between ‘financial hardships’ and ‘relational difficulties’ | | |
| Housing challenges | 3.05 (2.82, 3.28) | <0.001 |
| Financial hardships | 1.28 (1.08, 1.48) | <0.001 |
| Relational difficulties | 4.55 (4.32, 4.77) | <0.001 |
| Edu-employment issues | 1.92 (1.77, 2.06) | <0.001 |
| Interaction | -0.89 (-1.29, -0.49) | <0.001 |
| Model with the interaction between ‘housing challenges’ and ‘relational difficulties’ | | |
| Housing challenges | 3.31 (3.02, 3.59) | <0.001 |
| Financial hardships | 1.09 (0.91, 1.26) | <0.001 |
| Relational difficulties | 4.51 (4.28, 4.73) | <0.001 |
| Edu-employment issues | 1.92 (1.77, 2.07) | <0.001 |
| Interaction | -0.90 (-1.36, -0.44) | <0.001 |
| Model with the interaction between ‘housing challenges’ and ‘financial hardships’ | | |
| Housing challenges | 3.42 (3.11, 3.73) | <0.001 |
| Financial hardships | 1.31 (1.10, 1.51) | <0.001 |
| Relational difficulties | 4.28 (4.09, 4.47) | <0.001 |
| Edu-employment issues | 1.92 (1.77, 2.06) | <0.001 |
| Interaction | -0.94 (-1.37, -0.51) | <0.001 |

**NB.** All coefficients were estimated from multiple imputed multivariable linear regression models controlling for confounding factors including gender identity, age groups, Indigenous status, IRSAD decile, remoteness, and whether the young person reported speaking a language other than English at home. Only models with a p-value of interaction term lower than 0.001 were reported.

**Table S5**. Multivariable linear regression for PWI-SC (fully adjusted) with interaction terms.

|  | Coef (95% CI) | p-value |
| --- | --- | --- |
| Model with the interaction between ‘financial hardships’ and ‘relational difficulties’ | | |
| Housing challenges | -6.72 (-7.43, -6.02) | <0.001 |
| Financial hardships | -4.62 (-5.22, -4.02) | <0.001 |
| Relational difficulties | -11.50 (-12.17, -10.82) | <0.001 |
| Edu-employment issues | -7.38 (-7.82, -6.94) | <0.001 |
| Interaction | 2.40 (1.19, 3.61) | <0.001 |
| Model with the interaction between ‘housing challenges’ and ‘financial hardships’ | | |
| Housing challenges | -7.97 (-8.93, -7.01) | <0.001 |
| Financial hardships | -4.82 (-5.43, -4.21) | <0.001 |
| Relational difficulties | -10.77 (-11.34, -10.20) | <0.001 |
| Edu-employment issues | -7.37 (-7.81, -6.93) | <0.001 |
| Interaction | 3.16 (1.84, 4.47) | <0.001 |

**NB.** All coefficients were estimated from multiple imputed multivariable linear regression models controlling for confounding factors including gender identity, age groups, Indigenous status, IRSAD decile, remoteness, and whether the young person reported speaking a language other than English at home. Only models with a p-value of interaction term lower than 0.001 were reported.
